# *phenix.mr_rosetta*: molecular replacement and model rebuilding with *Phenix* and *Rosetta*

**DOI:** 10.1007/s10969-012-9129-3

**Published:** 2012-03-15

**Authors:** Thomas C. Terwilliger, Frank DiMaio, Randy J. Read, David Baker, Gábor Bunkóczi, Paul D. Adams, Ralf W. Grosse-Kunstleve, Pavel V. Afonine, Nathaniel Echols

**Affiliations:** 1 Los Alamos Institutes and BioScience Division, Los Alamos National Laboratory, Los Alamos, NM 87545 USA; 2 Department of Biochemistry, University of Washington, Seattle, WA 98195 USA; 3 Department of Haematology, Cambridge Institute for Medical Research, University of Cambridge, Cambridge, CB2 0XY UK; 4 Lawrence Berkeley National Laboratory, One Cyclotron Road, Bldg 64R0121, Berkeley, CA 94720 USA

**Keywords:** Molecular replacement, Automation, Macromolecular crystallography, *Rosetta*, *Phenix*

## Abstract

The combination of algorithms from the structure-modeling field with those of crystallographic structure determination can broaden the range of templates that are useful for structure determination by the method of molecular replacement. Automated tools in *phenix.mr_rosetta* simplify the application of these combined approaches by integrating *Phenix* crystallographic algorithms and *Rosetta* structure-modeling algorithms and by systematically generating and evaluating models with a combination of these methods. The *phenix.mr_rosetta* algorithms can be used to automatically determine challenging structures. The approaches used in *phenix.mr_rosetta* are described along with examples that show roles that structure-modeling can play in molecular replacement.

## Introduction

Molecular replacement [1] is an exceptionally powerful technique for the determination of structures of macromolecules. In molecular replacement a template structure serves as an initial model for the structure to be determined. The orientation and location of the template in the crystallographic cell are found by optimizing the agreement between measured structure factors and those calculated from the placement of the template. Then the placed template is used to estimate the crystallographic phases, allowing calculation of a preliminary electron density map. A new model is then built using this map as a guide.

Molecular replacement accounts for over 70% [2] of the structures in the Protein Data Bank (PDB, [3]). Despite this success, molecular replacement is limited to situations where a suitable template structure is available. The template must normally represent a large fraction (usually more than 50%) of the structure and have a core whose atomic coordinates are superimposable within approximately 1.5–2 Å root mean square deviation (rmsd) of the target structure [4].

There are two steps in molecular replacement where the availability of a sufficiently similar template is crucial. The first is at the stage of finding the orientation and location of the template structure in the asymmetric unit of the structure to be determined. If the template is too different from the structure to be determined, the correct location and orientation may not be identifiable.

The second step that requires a template sufficiently similar to the structure to be determined is the rebuilding of a correctly-placed model. It is not uncommon for molecular replacement to yield a solution that is unambiguous in its placement yet leads to an electron density map that does not give any useful clues as to how to improve the model. In such cases it is again not feasible to proceed with structure determination.

These restrictions on the divergence between template and structure to be determined, along with the wide use of molecular replacement, mean that any improvements in the starting templates for molecular replacement, in methods for finding the location and orientation of the template, in methods for obtaining accurate phases from a preliminary model, or in methods for rebuilding molecular replacement models can substantially increase the number of structures that can be determined by molecular replacement.

There have recently been many important advances in all these areas. Improved starting templates for molecular replacement have been obtained by judicious pruning of parts of models that are less likely to be correct [5, 6, 7], by creating ensembles of templates [8, 9], using normal mode analysis [10, 11], and by systematic searches using many or all of the proteins in the Protein Data Bank [12, 13]. Improved methods for finding the placement of the template include the use of likelihood in scoring of placements and the development of approximations to the likelihood function that are accurate yet much more rapid [14]. Improvements in methods for obtaining phase information from a preliminary model include developments in algorithms for creating maps that optimally show unmodeled density [15] and developments in density modification procedures that reduce model bias [16]. Improvements in model-building algorithms include the use of iteration between model-building, refinement and map calculation or density modification [17, 18, 19] and the development of methods that can be used at resolutions lower than 3 Å [20, 21, 22, 23, 24, 25, 26, 27].

A recent approach to obtaining improved templates for molecular replacement is to apply tools from the structure modeling field before or after placing the template in the crystallographic cell [28, 6, 29, 30]. The key idea in this approach is that crystallographic model-building and structure modeling use fundamentally different sources of information so that combining them can yield a more powerful approach to model-building than either alone.

Table 1 compares the algorithms and information used in crystallographic model-building and in structure modeling. Crystallographic model-building of macromolecules is based on interpretation of patterns of electron density. The presence of a polypeptide backbone, side-chains, and secondary structure are used directly in interpreting an electron density map in terms of an atomic model. In contrast, the core aspect of structure modeling is the use of specialized force fields capable of distinguishing a physically plausible model from one that is not. The algorithms in the structure modeling field are in optimal cases able to generate and refine structures with near-native conformations without the use of experimental information. For example Das and Baker [31] estimated that about one in six proteins under 100 amino acids in length can be modeled ab initio with sufficient accuracy for phasing by molecular replacement.

**Table 1:** Complementarity of model-building in macromolecular crystallography and in structure-modeling

Characteristic	Crystallographic model-building (*Phenix*)	Structure-modeling (*Rosetta)*
Optimization	Interpretation of patterns of density	Creating physically plausible models
Model-building approach	Density search for secondary structure	Ab initio modeling or homology modeling
Fragment libraries	3-residue fragment library	3- and 9-residue libraries
Model-building target	Fit to density	*Rosetta* force field (optional density term)
Refinement target	Structure-factor likelihood	*Rosetta* force field (optional density term)

Crystallographic model-building does make use of force fields as well. After model-building, crystallographic structures are refined using a combination of the agreement with crystallographic data and a simple set of geometric restraints. The restraints used in crystallographic model-building are normally much less sophisticated than the force fields used in the structure modeling field, however. They often do not include electrostatic or hydrogen bonding interactions for example. In contrast to refinement with force fields used in structure modeling, refinement of a structure with geometric restraints in the absence of crystallographic data typically is highly unlikely to converge to near-native conformations.

Qian et al. [29], Ramelot et al. [30], DiMaio et al. [28] and Mao et al. [6] have shown that *Rosetta* structure modeling can be used to improve homology models to make them more useful for finding their locations in a crystallographic cell, the first step in molecular replacement. Qian et al. [29] have shown that in some cases ab initio models created with *Rosetta* from sequence information alone can be sufficiently accurate to be useful in this step. DiMaio et al. [28] have shown further that augmentation of *Rosetta* structure modeling with pseudo-energy terms representing fit of model to electron density can greatly improve the rebuilding of models in the second key step of molecular replacement.

The procedures used by DiMaio et al. [28] for combining *Rosetta* structure modeling and crystallographic model-building require considerable manipulations and familiarity with both crystallographic and structure-modeling tools. To make the use of these procedures more accessible to a broader range of structural biologists, we developed software in the *Phenix* crystallographic computing environment [32] that provides simultaneous access to *Rosetta* structure modeling and *Phenix* crystallographic model-building [28]. This *phenix.mr_rosetta* software allows a user to identify suitable templates for molecular replacement available in the PDB, edit them to match the target sequence, optionally refine their structures with *Rosetta* prior to molecular replacement [29], carry out molecular replacement, and rebuild the resulting models with *Rosetta* [28] and *Phenix* autobuilding [19] algorithms. Alternatively the same software can begin with a partial or complete model already placed in a crystallographic cell and rebuild the model with *Rosetta* and *Phenix* autobuilding approaches. These procedures can be carried out using simple keyworded scripts that specify the input data and the procedures to be used. Here we describe the methods used in *phenix.mr_rosetta* and present examples that help show how the approach works.

## Methods

### Steps in molecular replacement and model rebuilding by *phenix.mr_rosetta*

The basic data required to run the *phenix.mr_rosetta* procedure consists of the sequence of the structure to be determined and the measured crystallographic structure-factor amplitudes for this structure. Additionally either a file from the *hhpred* server (http://toolkit.tuebingen.mpg.de/hhpred; [33]) listing similar proteins in the PDB and their alignments, or one or more templates edited to match the target sequence are required. For loop-building in *Rosetta*, files containing 3-residue and 9-residue fragments from the PDB tailored for the target protein are also required. These fragments can be obtained from the *Robetta* fragment server (http://robetta.bakerlab.org/fragmentsubmit.jsp) [34].

The overall procedure used in *phenix.mr_rosetta* consists of six steps. These are (1) downloading suitable templates and editing them to match the target sequence, (2) optional optimization of the models with *Rosetta* without using X-ray data, (3) placement of templates using molecular replacement, (4) refinement and calculation of density-modified electron density maps, (5) model rebuilding with *Rosetta* including density information, and (6) model rebuilding with *phenix.autobuild*.

Once the entire cycle of 6 steps has been carried out, a partially or completely built model may be obtained. If all chains in the model are found in the molecular replacement step (step 3) but the model is not fully rebuilt after carrying out these steps, then steps (4–6) of this procedure can be iterated to complete and further improve the resulting models. Alternatively, if some chains in the model are not found in molecular replacement, those that are found can be rebuilt in steps (4–6). Then the resulting model can be used as a fixed model for another molecular replacement attempt, and the resulting model can be rebuilt as before. The six steps are described in more detail below.

#### Downloading suitable templates and editing them to match the target sequence

The simplest starting point for *phenix.mr_rosetta* is a list of proteins in the PDB that are likely to have similar structures to the target protein. The hhpred server (http://toolkit.tuebingen.mpg.de/hhpred) [33] provides a rapid analysis of homologous sequences that are present in the PDB and lists these PDB entries along with their sequence alignments to the target structure. If the resulting summary file is supplied, *phenix.mr_rosetta* will use the tool *phenix.mr_model_preparation* to download a specified number of these PDB entries and edit them to match the sequence of the target protein. These edited templates can then either be the starting points for structure optimization by *Rosetta* or serve as search models for molecular replacement.

This simple procedure is limited to structures that can be represented by a single template from the PDB. Normally this means that it is suitable for structures with a single type of polypeptide chain. Structures that contain several different chains or chains that require several templates to be represented can be built with *phenix.mr_rosetta* but the initial molecular replacement steps must be carried out separately. The tool *phenix.mr_model_preparation* can be used to download and edit multiple templates and the molecular replacement tool *phenix.automr* can then be used to carry out molecular replacement with *Phaser* [14] to place and combine these templates. Then any number of the resulting potential molecular replacement solutions (placed models) can be used as the starting point for *phenix.mr_rosetta* beginning in step (4) below.

#### Optional optimization of the models with *Rosetta*

Once a template structure is available, *Rosetta* modeling tools [29] can optionally be applied to remodel the template. The information that is available for this remodeling is the sequence alignment between the template and the target molecule and the starting structure of the template. *Rosetta* can be used to rebuild the template, making its structure more compatible with the sequence of the target molecule and creating new chains for any gaps where the template did not match the target sequence. This process is carried out without reference to any crystallographic data. Normally 1,000–2,000 *Rosetta* models are created and the top-scoring model (based on the standard *Rosetta* energy function) is used as a search model in the molecular replacement step.

#### Placement of search models using molecular replacement

Once search models are available, molecular replacement is carried out using the crystallographic data along with each search model in turn. In cases where the size of the asymmetric unit of the crystal can accommodate more than one copy of the search model, the number of copies of the search model to be found can be specified, or *phenix.mr_rosetta* can try all plausible numbers of copies. If the number of copies to be found is a multiple of the number of copies of the template in its original crystallographic asymmetric unit, then the corresponding multimer of the template is tested in molecular replacement as well as the monomer. For example, if the template was a dimer in its original crystal form and four copies of the molecule can fit in the asymmetric unit of the target structure, then both the monomer and dimer of this template would be considered in separate runs of molecular replacement by *phenix.mr_rosetta*.

As there may be several search models and several numbers of copies to be tested, the entire molecular replacement step can produce a number of possible models. These models are rescored with the *Phaser* log-likelihood scoring procedure [9] using a fixed value of the estimated rmsd between template and target structure (typically using the smallest value of the estimated rmsd for all the search models considered). The best-scoring model or models are then considered as starting points for map calculation and *Rosetta* rebuilding.

#### Refinement and calculation of density-modified electron density maps

Once a potential molecular replacement solution is obtained, it is refined with *phenix.refine* [35] and the resulting model is used along with the experimental data to create a model-based density-modified electron density map with *Resolve* density modification [19]. If more than one copy of the template is present in the molecular replacement model, then non-crystallographic symmetry is included in the density modification procedure [36].

If the starting point for the entire procedure is a model already placed in the crystallographic cell, then this model is refined and a density-modified map is created in the same way. In this case the model can consist of any number of copies of any number of different chains. This allows the application of later steps in *phenix.mr_rosetta* to structures that are more complicated than those that can be described with a single sequence.

#### Model rebuilding with *Rosetta* including density information

Once a model has been placed in the crystallographic cell and a density map has been created, a *Rosetta* modeling procedure is carried out in which the *Rosetta* energy function is augmented with a term describing the fit of the model to the density [37, 28]. This *Rosetta* modeling procedure can rebuild existing segments of the model as well as build short loops (typically up to 8 residues in length) in gaps of the model. There can still be segments that are missing in the model, however. The resulting models with the best *Phaser* likelihood scores [9] are then refined with *phenix.refine* and used to create a new set of density-modified maps. These maps are averaged to yield a single averaged density-modified map. The refined *Rosetta* models are then rebuilt one more time with *Rosetta* using the fit to this averaged map in scoring and the best-scoring models are refined with *phenix.refine* and used as the starting point for *phenix.autobuild* automated model rebuilding.

In cases where more than one copy of a chain is present in the model, a single copy is supplied to *Rosetta* along with the density map corresponding to that chain. Then the resulting *Rosetta* model is copied to the locations of each of the copies in the original model to form a new *Rosetta*-based model with idealized non-crystallographic symmetry. In cases where more than one type of chain is present, one copy of each type of chain is supplied at a time to *Rosetta*. In this way any number of copies of any number of types of chains can be rebuilt with *Rosetta* including a density term.

#### Model rebuilding with *phenix.autobuild*

Model rebuilding is continued using *phenix.autobuild.* The starting points are the models rebuilt as described above with *Rosetta*, including a density term in the *Rosetta* energy. These models are rescored using the *Phaser* likelihood score [9]. The top models (typically 2) are then rebuilt with *phenix.autobuild* [19] based on the crystallographic data and the sequence of the target macromolecule. This automated model-building procedure uses the starting model and any non-crystallographic symmetry to create a density-modified map in the same way as in step (3) above. The density-modified map is used as the basis for crystallographic model-building and recombination of the newly-built model with the existing model, and the resulting model is refined using the crystallographic data [35]. The overall rebuilding procedure is iterated until the R-value comparing the crystallographic data with data expected from the model does not change substantially from cycle to cycle.

In the model-building process some polypeptide chain can be built in regions that are not represented in the *Rosetta* model used to start the autobuilding process. The sequences corresponding to such chains may be identified by the correspondence between the sequence of the target structure and the shapes of side chains visible in the electron density map along the polypeptide chain. However some chains may be built that cannot automatically be assigned to sequence. These are normally discarded if further cycles of *Rosetta* model-building are to be carried out as *Rosetta* model-building requires a knowledge of the sequence of the model to be rebuilt.

At the conclusion of autobuilding, the model with the lowest R-value and the corresponding density-modified map are saved. This model and map can be suitable for further rebuilding with semi-automated tools or re-used as the input for further cycles of *Rosetta* and *phenix.autobuild* rebuilding.

## Results and discussion

### Application of *phenix.mr_rosetta* to challenging structure determinations

Recently we have used a combination of *Rosetta* and *Phenix* to determine 13 new structures that had proven difficult or not possible to determine by a variety of other approaches [28]. The procedures used in *phenix.mr_rosetta* are automated versions of the procedures used in that work. Here we describe the application of *phenix.mr_rosetta* to two of these structures to illustrate how the combination of structure modeling and crystallographic model-building can enhance structure determination by molecular replacement.

#### Structure-modeling of an NMR model prior to molecular replacement

One of the structures determined by a combination of *Rosetta* modeling and *Phenix* autobuilding was the structure of the *radA* intein (structure #12 in [28, 38]). X-ray diffraction data were available to a resolution of 1.7 Å, and a dimer of the molecule is present in the asymmetric unit of the crystal in space-group *P2*_*1*_ *2*_*1*_ *2*_*1*_ *.* Additionally, an NMR model potentially suitable for use in molecular replacement was available (this NMR model was not a final model, but rather one that had been generated from NMR data using rapid automated procedures). Molecular replacement with the automatically-generated NMR model had not succeeded, but the structure could be determined by applying *Rosetta* structure modeling to the automatically-determined NMR model, choosing the best-scoring *Rosetta* model, and using that model in molecular replacement followed by *Phenix* autobuilding [28, 38].

**Fig. 1:**
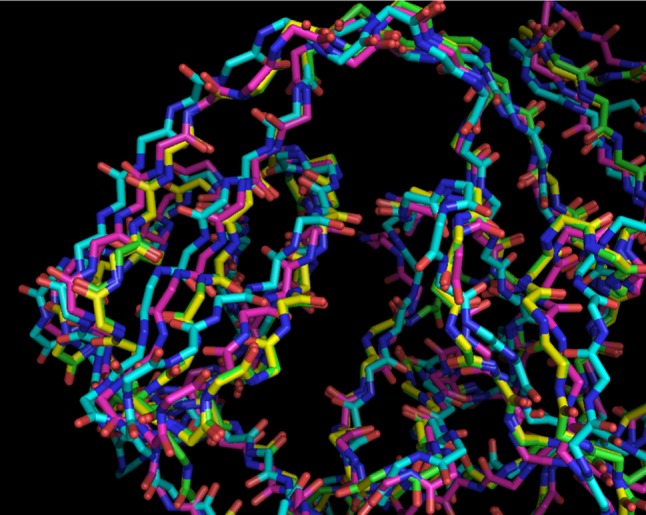
Comparison of models for the structure *radA* intein. The final refined structure [28] is shown in yellow. The NMR template is shown in *blue*. The best-scoring *Rosetta* model is in* purple*, and the *phenix.mr_rosetta* structure is in *green* (nearly superimposing on the final refined structure)

This structure determination can be reproduced with *phenix.mr_rosetta* by supplying the automatically-generated NMR model, the sequence of the protein, and the X-ray diffraction data, and specifying that the model is to be prerefined with *Rosetta* prior to molecular replacement. Figure 1 illustrates how the *Rosetta* refinement (without X-ray data) improves the automatically-generated NMR model sufficiently for it to be useful in molecular replacement. Figure 1 compares the final model of this structure (in yellow) with the NMR model (in blue), after superimposing the NMR model on the final model. The rmsd between the main-chain atoms of these models is about 2.1 Å (excluding residues 118–133 that are completely different), so it is not surprising that the automatically-generated NMR model is not successful in molecular replacement. For the 1,000 *Rosetta* models built in the *phenix.mr_rosetta* run, the mean value of this rmsd is 1.7 Å, with a range from 1.1 to 2.6 Å. Figure 1 shows the highest-scoring *Rosetta* model (in purple). This model is considerably better than an average *Rosetta* model, with an rmsd to the final structure of 1.5 Å (though not as accurate as the best *Rosetta* model). This improvement of the starting model from an rmsd of 2.1 to 1.5 Å is the critical step in the solution of this structure. Beginning with this highest-scoring *Rosetta* model, molecular replacement is successful (the top *Phaser* solution is correct), and refinement of the molecular replacement solution yields R and free R-values of 0.38 and 0.44. Subsequent rebuilding with *Rosetta* and *Phenix* autobuilding leads to a largely-correct model (the model in green in Fig. 1) with an R-value and free R-value of 0.24 and 0.27, respectively.

#### Structure-modeling with density to yield critical improvements in a placed model

A structure for which *Rosetta* modeling substantially aided crystallographic model-building is the protease *XMRV PR* [39], structure #6 in [28]. Efforts to determine this structure by standard molecular replacement approaches had failed, and the structure was determined by a combination of extensive molecular replacement and Rosetta modeling with electron density restraints using X-ray data collected to a resolution of 2 Å [39]. The structure was determined by creating a symmetric dimer from chain A of the HIV-1 protease structure *2hs1* [40] with a sequence identity of 30%. There is a dimer of *XMRV PR* in the asymmetric unit of the crystal. The location of a symmetric dimer from the template *2hs1* could be determined by molecular replacement, but the resulting model was too different from the template to yield a useful electron density map for rebuilding [28]. Rebuilding this model with *Phenix* autobuilding failed (with free R-value of 0.57).

**Fig. 2:**
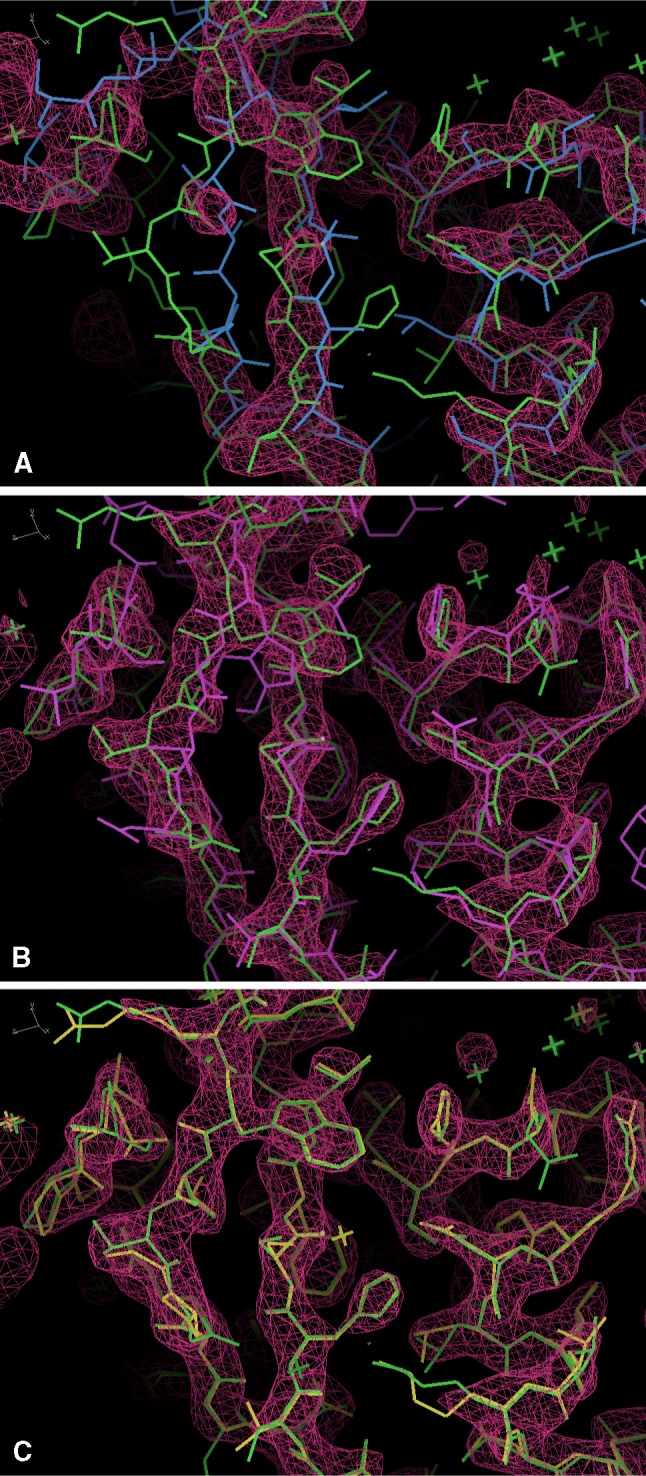
Models and maps for *XMRV PR* structure determination starting from a symmetric dimer placed by molecular replacement. An arbitrary region of the structure is shown that is generally representative of the overall maps and model. Maps are contoured at 1.5 σ. Figures generated with Coot [42]. **a** Placed template (*blue*) and final refined model [28, 39]; in *green*. The density-modified electron density map is based on refined placed template, including non-crystallographic symmetry in the density modification procedure. **b** Best-scoring *Rosetta* model (*purple*) created from the placed template and using the density map shown in **a**. The final refined model is shown in *green*. The averaged density-modified map created from the four best-scoring *Rosetta* models is shown. **c** Model produced by *phenix.autobuild* starting from the *Rosetta* model and averaged map shown in **b**

Figure 2a illustrates why this autobuilding failed. This figure shows the placed template (a symmetric dimer) from *2hs1* in blue, the final refined model of *XMRV PR* in green, along with the density-modified electron density map based on this placed template. This density map has a correlation of 0.56 to a map calculated from the final *XMRV PR* model. The map is difficult to interpret in many places and it is therefore not simple to improve the model. *Rosetta* modeling using the density map was able to improve the template considerably. Figure 2b shows the best-scoring *Rosetta* model (in purple), also along with the final refined model. The density-modified map obtained by averaging the density-modified maps from the top 4 best-scoring *Rosetta* models is shown. This map is substantially clearer than the one based on the placed template (it has a correlation of 0.82 to the final map) and allowed rebuilding of the best-scoring *Rosetta* model with *Phenix* autobuilding. At the end of this cycle of *phenix.mr_rosetta* building, the R-value and free R-value were 0.29 and 0.34, respectively, and the map correlation was 0.85 (Fig. 2c).

#### Application of *phenix.mr_rosetta* to 13 previously-solved structures

We tested the *phenix.mr_rosetta* tool by applying it to 13 structures previously solved using a combination of molecular replacement, structure-modeling and crystallographic model building [28]. Table 2 (column F) lists the free R-values of models obtained using *phenix.mr_rosetta* for each of these 13 structures. In most cases *phenix.mr_rosetta* was initiated with sequence alignments (listed with starting points of “sequence alignment” in Table 2), and in others (listed as “placed template”) the process was started after molecular replacement had been carried out. The structures in Table 2 are sorted according to the resolution of the data. For structures where high-resolution data (<2.5 Å) was available, the models obtained by *phenix.mr_rosetta* are quite accurate (with free R-values of 0.34 or better). For structures with lower-resolution data, *phenix.mr_rosetta* produced less-accurate models, but in all cases the maps obtained were of good or very good quality (map correlations to final refined structures ranged from 0.5 to 0.85). Overall, 11 of 13 of these datasets led to structures with free R-values of 0.42 or lower with *phenix.mr_rosetta*. The remaining two had free R-values of 0.44. Based on these results, it appears that the use of *phenix.mr_rosetta* would have been sufficient to solve all of these structures.

**Table 2:** Structure determinations with *phenix.mr_rosetta*

A	B	C	D	E	F	G	H
Structure	Resolution (Å)	Sequence identity (%)	NCS^a^ copies	Starting point^b^	*mr_rosetta* free R	*Autobuild* free R (from *mr_rosetta* placed templates)	*Autobuild* free R (from DiMaio et al. templates)
*radA intein*	1.7	100	2	NMR template	0.27	0.55	0.51
*cab55348*	1.9	31	1	Alignment file	0.28	0.32	0.52
*XMRV PR*	2.0	30	2	Alignment file^c^	0.34	0.37	0.57
*fk4430*	2.1	22	1	Alignment file	0.31	0.33	0.31
*thiod*	2.1	22/15	1	Placed template	0.29	0.51	0.56
*bfr258e*	2.2	19	2	Alignment file	0.30	0.29	0.29
*niko*	2.5	27	2	Alignment file	0.28	0.31	0.34
*estan*	2.5	18	1	Alignment file	0.28	0.55	0.55
*fj6376*	2.7	21	4	Alignment file	0.30	0.30	0.30
*pc02153*	2.8	29	1	Alignment file	0.44	0.45	0.54
*pc0265*	2.9	29	2	Placed template	0.40	0.42	0.46
*tirap*	3.0	22	1	Alignment file	0.44	0.44	0.46
*hp3342*	3.2	20	1	Placed template	0.41	0.51	0.50

The names of the structures and correspondences with the structure numbers used in [28] are those provided at http://www.phenix-online.org/phenix_data/terwilliger/rosetta_2011/ except that the structure *radA* intein is alternatively referred to as *ag9603a*

^a^NCS copies is the number of copies of the molecule in the asymmetric unit of the crystal related by non-crystallographic symmetry

^b^The starting point for each structure determination with *phenix.mr_rosetta* (column F) was a sequence alignment obtained with the hhpred server (http://toolkit.tuebingen.mpg.de/hhpred; [33]; indicated as “alignment file” in column E), or an NMR model (indicated with “NMR template”), or an edited template structure, placed in the correct position by *Phaser* [14] as used in DiMaio et al. [28]; indicated with “placed template”. In column G the free R-values obtained by rebuilding the placed model from the corresponding *phenix.mr_rosetta* analysis with *phenix.autobuild* are shown. In column H the free R-values obtained by rebuilding the placed models used in DiMaio et al. [28] are shown (the *phenix.autobuild* runs are different and consequently the free R-values differ somewhat from those reported in [28])

^c^The structure *XMRV PR* could be solved either automatically with *phenix.mr_rosetta* beginning with just the sequence alignment (in column F of this Table) or beginning with a placed symmetric dimer (as in Fig. 2). The automated *phenix.mr_rosetta* structure determination beginning with a sequence alignment (column F) yielded a molecular replacement solution using the dimer of *2hs1* [40] and this molecular replacement solution could be rebuilt either with (column F) or without (column G) *Rosetta* modeling. The symmetric dimer molecular replacement solution shown in Fig. 2 could only be rebuilt using *Rosetta* modeling with density (poor free R value for rebuilding with *phenix.autobuild* alone shown in column H)

These 13 structures and their experimental data have been examined quite extensively [28] and many different approaches for structure determination have been applied to each of them. In previous work the key question was how much information was contributed by the use of *Rosetta* modeling. To answer this question, the comparisons among methods all began with templates placed in the crystallographic unit cell using *Phaser* molecular replacement, and the effectiveness of each method in improving these placed models was examined [28]. Those comparisons showed that for two of the structures (*radA intein* and *pc0265*), *Rosetta* modeling was essential for the first step in molecular replacement to succeed. For 6 additional structures (*XMRV PR, thiod, pc02153, tirap,* *hp3342* and *estan*) *Rosetta* modeling with density after molecular replacement yielded substantially better models than the other methods tried. The next-best method for these 6 structures consisted of deformable elastic network (DEN) refinement [41] followed by *Phenix* autobuilding. For the final 5 structures (*fk4430, bfr258e, niko, fj6376* and *cab55348*) several methods, including *Rosetta* modeling with density, could be used to determine the structures.

Table 2 (columns G and H) lists the free R-values obtained by using *phenix.autobuild* (without including *Rosetta* structure-modeling) to rebuild the templates placed with *phenix.mr_rosetta* (column G) or the templates used in DiMaio et al. [28]. Rebuilding the templates used in the previous analysis [28], with *phenix.mr_rosetta* (column H) gave results similar to those reported previously [28]. In only 4 of 13 cases did autobuilding yield free R-values of 0.42 or better. This shows the need for other approaches such as *Rosetta* modeling to improve these models before crystallographic autobuilding could be used.

Some of the template placements found in the molecular replacement step by *phenix.mr_rosetta* were closer to the final structures than those used in DiMaio et al. [28]. The molecular replacement searches carried out by *phenix.mr_rosetta* in Table 2 (column F) were in some cases quite extensive. Some used as many as 13 starting templates. Others tested various possibilities for the number of copies in the asymmetric unit or various possibilities for the number of chains from the deposited structures used as templates in the molecular replacement search. The result of the extensive search approach can be seen from column G of Table 2, in which the templates placed by *phenix.mr_rosetta* were used directly in autobuilding (without the use of *Rosetta*)*.* Using *phenix.autobuild* with these templates, 7 of the 13 structures could be determined with free R-values of 0.42 or better. This result is consistent with the known utility of extensive searches with a variety of molecular replacement templates (e.g., [12, 13].

## Conclusions

The combination of structure modeling with *Rosetta* and crystallographic model-building techniques can substantially increase the range of templates that are suitable for molecular replacement [28]. The automated tools in *phenix.mr_rosetta* simplify the application of these combined approaches by integrating the *Phenix* and *Rosetta* algorithms and by systematically generating and evaluating models with a combination of these methods. As demonstrated here, the *phenix.mr_rosetta* algorithms can be used to automatically determine some of the most challenging structures determined by manual combination of molecular replacement and *Rosetta.*

The *Rosetta* and *Phenix* tools available in *phenix.mr_rosetta* can address each of the steps in molecular replacement that can fail because of lack of a template that is close enough to the target molecule. In cases where the template is so different that it cannot be successfully placed in the crystallographic cell, *phenix.mr_rosetta* can use *Rosetta* modeling to improve the template. As shown above for the *radA* intein structure, this improvement can be sufficient to allow molecular replacement and the subsequent rebuilding. In cases where the template is similar enough to the target structure for placement of the model, but too different for model rebuilding, *phenix.mr_rosetta* can use *Rosetta*, along with an electron density map, to improve the placed template. This was illustrated with the *XMRV PR* structure determination described above. The key step in this structure determination was the slight improvement in the model obtained by *Rosetta* rebuilding with density. Without this improvement, the model was too poor to yield a map that is interpretable, but with it the map was improved enough to allow rebuilding. This is the essence of the combination of *Rosetta* modeling with crystallographic model-building. The combination allows borderline cases, which are apparently quite frequent, to be solved by incorporating some complementary information from the *Rosetta* modeling that moves the starting model closer to the target structure.

The approaches used in *phenix.mr_rosetta* are likely to be applicable not only to molecular replacement, as in the examples described here, but also to other situations where model rebuilding is challenging but the sequence of the model being built is known. For example, it is not uncommon for an experimental structure determination to lead to a mostly-complete model that is outside the range of convergence of current refinement procedures. This can occur if the resolution is low or if the quality of the experimental electron density map is too poor to build an accurate model. The sequence associated with the model might be known or a limited number of possibilities for sequence assignment might be obtained. In such cases *phenix.mr_rosett*a tools may be useful in rebuilding the models, bringing in information from structure-modeling to improve the quality of the models and the resulting electron density maps, and ultimately leading to more complete and accurate models.

## References

[1] Rossmann MG (1972). The molecular replacement method.

[2] Evans P, McCoy A (2008). An introduction to molecular replacement. Acta Cryst.

[3] Berman HM, Westbrook J, Feng Z, Gilliland G, Bhat TN, Weissig H, Shindyalov IN, Bourne PE (2000). The protein data bank. Nucleic Acids Res.

[4] Chen YW, Dodson EJ, Kleywegt GJ (2000). Does NMR mean “not for molecular replacement”? using NMR-based search models to solve protein crystal structures. Structure.

[5] Bunkoczi G, Read RJ (2010). Improvement of molecular-replacement models with Sculptor. Acta Cryst.

[6] Mao B, Guan R, Montelione GT (2011). Improved technologies now routinely provide protein NMR structures useful for molecular replacement. Structure.

[7] Schwarzenbacher R, Godzik A, Grzechnik SK, Jaroszewski L (2004). The importance of alignment accuracy for molecular replacement. Acta Cryst D.

[8] Keegan RM, Long F, Fazio VJ, Winn MD, Murshudov GN, Vagin AA (2011). Evaluating the solution from MrBUMP and BALBES. Acta Cryst.

[9] Read RJ (2001). Pushing the boundaries of molecular replacement with maximum likelihood. Acta Cryst D.

[10] Delarue M (2008). Dealing with structural variability in molecular replacement and crystallographic refinement through normal-mode analysis. Acta Cryst.

[11] Kidera A, Go N (1992). Normal mode refinement—crystallographic refinement of protein dynamic structure. 1. Theory and test by simulated diffraction data. J Mol Biol.

[12] Long F, Vagin A, Young P, Murshudov GN (2008). BALBES: a molecular replacement pipeline. Acta Cryst.

[13] Stokes-Rees I, Sliz P (2010). Protein structure determination by exhaustive search of Protein Data Bank derived databases. Proc Natl Acad Sci USA.

[14] McCoy AJ, Grosse-Kunstleve RW, Adams PD, Winn MD, Storoni LC, Read RJ (2007). Phaser crystallographic software. J Appl Cryst.

[15] Roversi P, Blanc E, Vonrhein C, Evans G, Bricogne G (2000). Refinement of severely incomplete structures with maximum likelihood in BUSTER-TNT. Acta Cryst.

[16] Terwilliger TC (2004). Using prime-and-switch phasing to reduce model bias in molecular replacement. Acta Cryst.

[17] Langer G, Cohen SX, Lamzin VS, Perrakis A (2008). Automated macromolecular model building for X-ray crystallography using ARP/wARP version 7. Nat Protoc.

[18] Perrakis A, Morris R, Lamzin VS (1999) Automated protein model building combined with iterative structure refinement. Nature Struct Biol 6:458–463 10.1038/8263 10331874

[19] Terwilliger TC, Grosse-Kunstleve RW, Afonine PV, Moriarty NW, Zwart PH, Hung LW, Read RJ, Adams PD (2008). Iterative model building, structure refinement and density modification with the PHENIX AutoBuild wizard. Acta Cryst D.

[20] Baker ML, Ju T, Chiu W (2007). Identification of secondary structure elements in intermediate resolution density maps. Structure.

[21] Cowtan K (2006). The Buccaneer software for automated model building. Acta Cryst.

[22] DiMaio F, Kondrashov DA, Bitto E, Soni A, Bingman CA, Phillips GN, Shavlik JW (2007). Bioinformatics.

[23] Ioerger TR, Sacchettini JC (2003). TEXTAL system: artificial intelligence techniques for automated protein model building. Methods Enzymol.

[24] Levitt DG (2001). A new software routine that automates the fitting of protein X-ray crystallographic electron density maps. Acta Cryst.

[25] Oldfield TJ (2002). Pattern-recognition methods to identify secondary structure within X-ray crystallographic electron-density maps. Acta Cryst.

[26] Oldfield TJ (2003). Automated tracing of electron density maps of proteins. Acta Cryst.

[27] Terwilliger TC (2010). Rapid model-building of alpha-helices in electron density maps. Acta Cryst.

[28] DiMaio F, Terwilliger TC, Read RJ, Wlodawer A, Oberdorfer G, Wagner U, Valkov E, Alon A, Fass D, Axelrod HL, Das D, Vorobiev SM, Iwai H, Pokkuluri PR, Baker D (2011). Improving molecular replacement by density and energy guided protein structure optimization. Nature.

[29] Qian B, Raman S, Das R, Bradley P, McCoy AJ, Read RJ, Baker D (2007). High resolution structure prediction and the crystallographic phase problem. Nature.

[30] Ramelot TA, Raman S, Kuzin AP, Xiao R, Ma LC, Acton TB, Hunt JF, Montelione GT, Baker D, Kennedy MA (2009). Improving NMR protein structure quality by Rosetta refinement: a molecular replacement study. Proteins.

[31] Das R, Baker D (2009). Prospects for de novo phasing with de novo protein models. Acta Cryst.

[32] Adams PD, Afonine PV, Bunkoczi G, Chen VB, Davis IW, Echols N, Headd JJ, Hung LW, Kapral GJ, Grosse-Kunstleve RW, McCoy AJ, Moriarty NW, Oeffner R, Read RJ, Richardson DC, Richardson JS, Terwilliger TC, Zwart PH (2010). PHENIX: a comprehensive Python-based system for macromolecular structure solution. Acta Cryst.

[33] Söding J (2005). Protein homology detection by HMM–HMM comparison. Bioinformatics.

[34] Chivian D, Kim DE, Malmstrom L, Bradley P, Robertson T, Murphy P, Strauss CEM, Bonneau R, Rohl CA, Baker D (2003). Automated prediction of CASP-5 structures using the Robetta server. Proteins.

[35] Afonine PV, Grosse-Kunstleve RW, Adams PD (2005) The Phenix refinement framework. CCP4 newsl. 42, contribution 8

[36] Terwilliger TC (2002). Statistical density modification with non-crystallographic symmetry. Acta Cryst.

[37] DiMaio F, Tyka MD, Baker ML, Chiu W, Baker D (2009). Refinement of protein structures into low-resolution density maps using Rosetta. J Mol Biol.

[38] Lyskowski A, Oeemig JS, Jaakonen A, Rommi K, DiMaio F, Zhou D, Kajander T, Baker D, Wlodawer A, Goldman A, Iwaï H (2011). Cloning, expression, purification, crystallization and preliminary X-ray diffraction data of the Pyrococcus horikoshii RadA intein. Acta Cryst.

[39] Li M, DiMaio F, Zhou D, Gustchina A, Lubkowski J, Dauter Z, Baker D, Wlodawer A (2011). Crystal structure of XMRV protease differs from the structures of other retropepsins. Nat Struct Mol Biol.

[40] Kovalevsky AY, Liu F, Leshchenko S, Ghosh AK, Louis JM, Harrison RW, Weber IT (2006). Ultra-high resolution crystal structure of HIV-1 protease mutant reveals two binding sites for clinical inhibitor TMC114. J Mol Biol.

[41] Schröder G, Levitt M, Brünger AT (2010). Super-resolution biomolecular crystallography with low-resolution data. Nature.

[42] Emsley P, Lohkamp B, Scott WG, Cowtan K (2010) Features and development of Coot. Acat Cryst D66:486–501 10.1107/S0907444910007493 PMC2852313 20383002

